# Autocrine Prostaglandin E_2_ Signaling Promotes Tumor Cell Survival and Proliferation in Childhood Neuroblastoma

**DOI:** 10.1371/journal.pone.0029331

**Published:** 2012-01-19

**Authors:** Agnes Rasmuson, Anna Kock, Ole Martin Fuskevåg, Björn Kruspig, Jaione Simón-Santamaría, Vladimir Gogvadze, John Inge Johnsen, Per Kogner, Baldur Sveinbjörnsson

**Affiliations:** 1 Childhood Cancer Research Unit, Department of Women's and Children's Health, Karolinska Institutet, Stockholm, Sweden; 2 Department of Clinical Pharmacology, University Hospital of North Norway, Tromsø, Norway; 3 Division of Toxicology, Institute of Environmental Medicine, Karolinska Institutet, Stockholm, Sweden; 4 Vascular Biology Research Group, Faculty of Medicine, University of Tromsø, Tromsø, Norway; 5 Division of Immunology, Faculty of Health Sciences, University of Tromsø, Tromsø, Norway; University of Navarra, Spain

## Abstract

**Background:**

Prostaglandin E_2_ (PGE_2_) is an important mediator in tumor-promoting inflammation. High expression of cyclooxygenase-2 (COX-2) has been detected in the embryonic childhood tumor neuroblastoma, and treatment with COX inhibitors significantly reduces tumor growth. Here, we have investigated the significance of a high COX-2 expression in neuroblastoma by analysis of PGE_2_ production, the expression pattern and localization of PGE_2_ receptors and intracellular signal transduction pathways activated by PGE_2_.

**Principal Findings:**

A high expression of the PGE_2_ receptors, EP1, EP2, EP3 and EP4 in primary neuroblastomas, independent of biological and clinical characteristics, was detected using immunohistochemistry. In addition, mRNA and protein corresponding to each of the receptors were detected in neuroblastoma cell lines. Immunofluorescent staining revealed localization of the receptors to the cellular membrane, in the cytoplasm, and in the nuclear compartment. Neuroblastoma cells produced PGE_2_ and stimulation of serum-starved neuroblastoma cells with PGE_2_ increased the intracellular concentration of calcium and cyclic AMP with subsequent phosphorylation of Akt. Addition of 16,16-dimethyl PGE_2_ (dmPGE_2_) increased cell viability in a time, dose- and cell line-dependent manner. Treatment of neuroblastoma cells with a COX-2 inhibitor resulted in a diminished cell growth and viability that was reversed by the addition of dmPGE_2_. Similarly, PGE_2_ receptor antagonists caused a decrease in neuroblastoma cell viability in a dose-dependent manner.

**Conclusions:**

These findings demonstrate that PGE_2_ acts as an autocrine and/or paracrine survival factor for neuroblastoma cells. Hence, specific targeting of PGE_2_ signaling provides a novel strategy for the treatment of childhood neuroblastoma through the inhibition of important mediators of tumor-promoting inflammation.

## Introduction

Inflammatory cells and mediators are critical components of the tumor microenvironment. Many cancer cells have adapted inflammatory signaling molecules as autocrine and/or paracrine survival factors. Arachidonic acid-derived lipid mediators are very potent signaling molecules that are important in the inflammatory process and implicated in tumorigenesis. Conversion of arachidonic acid by the cyclooxygenase (COX) enzymes results in the production of prostaglandins and thromboxane. A large body of evidence has shown that COX-2 is often highly expressed in adult cancers of epithelial origin, and has been implicated in resistance to apoptosis, promotion of proliferation, increased tumor invasiveness and angiogenesis as well as decreased immunosurveillance [1]. Neuroblastoma, an embryonic tumor of early childhood, is enriched in arachidonic acid, and expresses high levels of COX-2 [2], [3]. Neuroblastoma arises from immature cells of the developing sympathetic nervous system, with primary tumors in the adrenal gland medulla or in paravertebral ganglia. The tumors exhibit very heterogeneous clinical behaviour with some congenital tumors spontaneously regressing even without any treatment whereas the majority of neuroblastoma patients present with aggressive metastatic tumors with poor prognosis despite very intensive therapy [4]. Therapeutic inhibition of the COX enzymes in neuroblastoma induces apoptosis, suppresses tumor growth, reduces angiogenesis and potentiates the toxic effect of cytostatic drugs [3], [5]–[7]. Inhibition of this pathway may represent a novel treatment strategy for neuroblastoma patients not cured today. However, clinical studies have raised concerns about the potential adverse side effects of NSAIDs in adults [8]. Also, COX inhibitors have shown to possess off-target effects that contribute to cancer inhibition [9]. Therefore, further investigation of a high COX-2 expression in neuroblastoma, and the possibility of a more specific targeting of this pathway is highly warranted.

Upon cellular stimuli arachidonic acid is released from membrane phospholipids by cytosolic phospholipase A_2_ (cPLA_2_). Arachidonic acid is then converted to prostaglandin H_2_ (PGH_2_) in a two-step reaction catalysed by either of the two COX isoforms, the constitutively active COX-1 or the inducible COX-2. PGH_2_ is further metabolized into the different prostaglandins by specific synthases [1]. Newly formed PGE_2_ can either act on receptors located near their site of synthesis or be transported out of the cell to act in an autocrine or paracrine manner [1], [10]. PGE_2_ exerts its effects by interacting with a subfamily of four distinct G-protein-coupled receptors (GPCR) designated EP1, EP2, EP3 and EP4 [11]. The EP1 receptor causes upon stimulation an increase of intracellular Ca^2+^. The EP2 and EP4 receptors are coupled to adenylate cyclase through a Gαs protein, increasing the cyclic adenosine monophosphate (cAMP) level. The EP3 receptor has several splice variants capable of coupling to different G-proteins thereby contributing to the wide spectrum of EP3 actions. However, the majority of EP3 isoforms couple to Gαi inhibiting adenylate cyclase and the production of cAMP [11].

In the present study, we have assessed the expression of the PGE_2_ receptors in neuroblastoma, and the role of PGE_2_ signaling in neuroblastoma survival and proliferation.

## Materials and Methods

### Human tissue samples

Neuroblastoma tumor tissue was obtained during surgery and stored in −80°C. Tissue samples from 28 neuroblastoma patients from all clinical stages [4] and different biological subsets were analyzed [12]. Ethical approval was obtained from the Karolinska University Hospital Research Ethics Committee (Approval no. 2009/1369-31/1 and 03-736). Informed consent for using tumor samples in scientific research was provided by parents/guardians. In accordance with the approval from the Ethics Committee the informed consent was either written or verbal. When verbal or written assent was not obtained the decision was documented in the medical record.

### Immunohistochemistry

Formalin-fixed and paraffin-embedded tissue slides were deparaffinized in xylol and rehydrated in graded alcohols. For antigen retrieval, slides were boiled in a sodium citrate buffer (pH 6.0) for 10 min, in a microwave oven. After blocking in 1% bovine serum albumin (BSA) for 20 min, the tissue sections were incubated with primary antibody overnight, against EP1, EP2, EP3 and EP4, respectively (Cayman Chemical, Ann Arbor, MI, USA) diluted 1∶250 in 1% BSA/PBS. Thereafter slides were rinsed in PBS and endogenous peroxidases were blocked in 0.3% H_2_O_2_ for 10 min. As a secondary antibody, anti-rabbit-horseradish peroxidase (HRP) SuperPicTure Polymer detection kit was used (Invitrogen, Paisley, UK). All slides were counterstained with haematoxylin. To control for non-specific binding, antibody specific blocking peptides and isotype-matched controls were used.

### Chemicals and solutions

Celecoxib was from Pfizer (Täby, Sweden). AH6809, AH23848, 16,16-dimethyl PGE_2_ (dmPGE_2_), PGE_2_, PGD_2_ and PGE_2_-d_4_ were purchased from Cayman Chemical. L-161,982 and SC-51322 were obtained from BioMol (Plymouth Meeting, PA, USA). ONO-8713 and ONO-AE3-240 were a generous gift from Ono Pharmaceutical (CO., Ltd., Osaka, Japan). Arachidonic acid was purchased from Nu-Check Prep (Elysian, MN, USA). 3-(4,5-dimethylthiazol-2-yl)-2,5-diphenyltetrazoleum bromide (MTT) was purchased from Sigma-Aldrich (Stockholm, Sweden). Analytical grade *n*-hexane, ethyl acetate, ammonium acetate and HPLC grade methanol were supplied by Merck (Darmstadt, Germany). 2,6-Di-tert-butyl-4-methylphenol (BHT) and citric acid were purchased from Fluka (Sigma-Aldrich).

### Cell lines

The human neuroblastoma cell lines, SH-SY5Y, SK-N-FI, SK-N-SH, SK-N-BE(2), SK-N-AS, SK-N-DZ and IMR-32 were cultured as previously described [3]. The human myelocytic cell line, U-937 (ATCC, Boras, Sweden), was grown in PRMI using the same supplements as mentioned above.

### Reverse transcriptase-polymerase chain reaction

RNA was extracted from cells using TRIZOL reagent (Life Technologies Inc.,Carlsbad, CA, USA) according to the manufacturer's protocol, and cDNA was synthesized from 2.0 µg of RNA using a SuperScript Preamplification Kit (Life Technologies Inc.). The PCR was performed in 50 µL of reaction mixture containing 4 µl cDNA for EP1-4, and 2 µl for β-Actin (for SK-N-SH 4 µL was used), 1 unit of DyNAzymeII Recombinant DNA (FinnzymesOy, Espoo, Finland) and 1 µM of each primer. Samples prepared for detection of EP1-3 were heated for 5 min at 94°C and amplified for 40 cycles of 30 s at 94°C, 1 min at 62°C, and 1 min at 72°C, and finally for 10 min at 72°C. For the detection of EP4, samples were amplified for 40 cycles of 45 s at 94°C, 1 min at 66°C and 45 s at 72°C, while for β-actin the amplification was performed in 40 cycles of 1 min at 94°C, 1 min at 55°C and 1 min at 72°C. Primer sequences were as follows: EP1 forward: 5′-TGGGCCAGCTTGTCGGTA-3; and reverse: 5-AGGGCCACCAACACCAG-3′; EP2 forward: 5′-TGGGTCTTTGCCATCCTT-3; and reverse: 5-TCCGACAACAGAGGACTG-3′; EP3 forward: 5′-CAGCTTATGGGGATCATG-3′; and reverse: 5-TCCGTG TGTGTCTTGCAG-3; EP4 forward: 5- TCGCGCAAGGAGCAGAAGGAGACG-3; and reverse: 5′-GGACGGTGGCGAGAATGAGGAAGG-3′. β-Actin forward: 5′- TGACGGGGTCACCCACACTGTGCCCATCTA-3′; and reverse: 5′-ACTCGTCATACTCCTGCTTGCTGATCCA-3′. PCR amplifications were performed in a PTC-200 Peltier Thermal Cycler (MJ Research Inc. Waltham, MA, USA). PCR products were analysed by agarose gel electrophoresis and photographed under UV light.

### Western blot analysis

For the detection of EP1-4, cells were lysed in RIPA buffer (Cell Signaling, Beverly, MA, USA) containing protease inhibitors (Roche Diagnostics GmbH, Mannheim Germany). Equal amounts of protein were separated by SDS-PAGE (Lonza, In vitro), transferred to nylon membranes (Millipore, Sundbyberg, Sweden) and probed with antibodies against EP1, EP2, EP3, EP4 (Cayman Chemical) or β-actin (Sigma-Aldrich), respectively. Anti-rabbit IgG conjugated with HRP (Cell Signaling) was used as a secondary antibody and Pierce Super Signal (Pierce, Rockford, IL, USA) was used for detection. The experiments were repeated twice. To investigate activation of the Akt signaling pathway by PGE_2_, SK-N-BE(2) and SK-N-SH cells were incubated in RPMI supplemented with L-glutamine, penicillin G and streptomycin, for 24 h before the addition of dmPGE_2_. After 1, 2, 4, 6, 12 or 24 h, cells were trypsinized, washed in cold PBS and lysed in RIPA buffer supplemented with protease inhibitors, and phosphatase inhibitors, 1 mmol/L NaF and 1 mmol/L NaO_3_V_4_. Membranes were probed with antibodies against phospho-Akt (ser473, Cell Signaling), Akt, or β-actin, respectively. Anti-rabbit-HRP served as a secondary antibody and a Pierce Super Signal was used for detection. Each experiment was repeated three times.

### Immunofluorescence

SH-SY5Y cells were grown on fibronectin coated chamber slides (Nunc, Roskilde, Denmark) for 24 h. Cultures were washed, and fixed with 2% paraformaldehyde for 15 min, followed by 70% cold methanol for 5 min. After washing with PBS, sections were incubated overnight at 4°C with rabbit-anti EP1-4 antibodies, respectively. After rinsing in PBS, cells were incubated with secondary antibodies conjugated with Alexa 488 (Invitrogen), mounted with medium containing DAPI and analysed in fluorescence microscopy. Image J software was used to merge the pictures (NIH, Bethesda, USA).

### Liquid-liquid extraction and LC-MS/MS analysis

Cells were cultured in a clear OptiMEM medium (Gibco, Paisley, UK) supplemented with 20% fetal bovine serum, L-glutamine, penicillin G, and streptomycin, both with or without 40 µM of arachidonic acid, and 10 ng/mL of IL-1β (R&D Systems, Abingdon, UK). Cells were harvested, washed and resuspended in Ca^2+^ and Mg^2+^ free-PBS supplemented with protease inhibitors, and sonicated three times for 10 s on ice. The homogenates were further incubated in 80 µM of arachidonic acid for 30 min at 37°C. Samples, standards and controls were extracted as previously described [13] in 4.5 ml polypropylene tubes (Sarstedt, Germany). Stock solutions were prepared in methanol to obtain a concentration of 1404 µmol/L for PGE_2_ and PGD_2_, and standard samples were prepared by dilution of the stock solution in PBS at a concentration ranging from 1–512 nM. Quality control samples were prepared in the same manner. The residue was then reconstituted in an 80 µl mobile phase and analyzed on a Waters Acquity UPLC (Waters Corp., Milford, MA, USA) interfaced to a Waters Quattro Premier XE tandem quadrupole mass spectrometer (Waters Corp). The system was controlled by MassLynx version 4.1. The chromatography was performed on a 2.1×100 mm Waters Acquity HSS T3 (C_18_) UPLC column maintained at 50°C with a programmed gradient from solvent A (methanol/water/ammonium acetate of 10/90/2 mM, v/v/concentration) to B (methanol/water/ammonium acetate 90/10/2 mM, v/v/concentration) at 0.4 ml/min to resolve the PGE_2_ from PGD_2_. The flow was diverted to waste before and after the analytes of interest. The mass spectrometer was operated in a negative electrospray ion mode, and the spray voltage was 3 kV. The sample injection volume was 15 µl and the injection interval was 7 min. The autosampler temperature was 5°C; desolvation gas temperature 340°C; source temperature 120°C; desolvation gas flow 900 L/h; cone gas flow 40 L/h; collision gas pressure 3.5×10^−3^ mBar (argon); ion energies, 0.9 V for both quadrupoles. For quantitative analysis of PGE_2_, the following MRM transitions were used: *m/z* 351→315 (quantification ion), 351→271 (qualifier ion) and 351→315 (qualifier ion). MRM transition *m/z* 355→319 and 355→275 were used for the internal standard. The dwell time was set to 20 ms for each transition. The method demonstrated a good linearity and reproducibility with a correlation coefficient (r^2^) of >0.99 and a coefficient of variation of <10%.

### Intracellular calcium mobilization

The intracellular mobilization of calcium in response to dmPGE_2_ was visualized by the fluorescent calcium dye Fluo-4/AM (Invitrogen) and measured using a confocal laser scanning microscope (Zeiss LSM 510 META). SK-N-SH (4×10^5^ cells) cells were seeded on cover slips in Petri dishes and cultivated overnight before incubation for 30 min at 37°C in Krebs-Ringer solution (119 mM NaCl, 2.5 mM KCl, 1.0 mM NaH_2_PO_4_ (monobasic), 2.5 mM CaCl_2_·2H_2_O,1.3 mM MgCl_2_·6H_2_O, 20 mM Hepes, 11 mM D-glucose C_6_H_12_O_6_, pH 7.4) containing 5 µM Fluo-4/AM. Thereafter, cells were washed with Krebs-Ringer solution and subsequently examined with a confocal laser scanning microscope. Before 1 µM of dmPGE_2_ was bath-applied a base line was determined, with or without 2 mM of EGTA (Sigma-Aldrich), for 5 min for each sample that was analysed. Images were processed and analysed using the Image J software.

### Cyclic AMP

The intracellular level of cAMP was determined using a cAMP EIA kit (Cayman Chemical). SK-N-SH cells (1.5×10^6^) were seeded in 10 cm^2^ Petri dishes and cultivated for 48 h. Cells were grown in RPMI supplemented with L-glutamine, penicillin G, and streptomycin overnight before the addition of 1 µM dmPGE_2_ for 2.5, 5, 10 or 20 min, or 10 µM forskolin (Enzo, Plymouth Meeting, PA, USA) for 10 min. As a negative control, cells were pre-treatment with 10 µM NF 449 (Santa Cruz Biotechnology, Santa Cruz, CA, USA) for 60 min before the addition of dmPGE_2_ for 10 min. The plates were rinsed with PBS, and lysed using 500 µL 0.1 M HCl for 20 min at room temperature. The plates were scraped and the lysates were collected and centrifuged. The supernatants were acetylated and used for intracellular cAMP determination according to the manufacturer's instructions.

### Cell viability assay

The effect of dmPGE_2_ on neuroblastoma cell viability was investigated using the MTT-assay [14]. SK-N-BE(2) (3×10^3^ cells/well) and SK-N-SH (2×10^4^ cells/well) cells were seeded in 96-well plates. After 24 h, the medium was changed to RPMI supplemented with L-glutamine, penicillin G, and streptomycin. After 24 h, an increasing concentration of dmPGE_2_ was added and the cells were further incubated for 24, 48, 72 and 96 h, respectively before subjected to MTT-assay. The medium was changed every second day and the mean values of optical density measurements of six separate wells were calculated. To determine the effect of dmPGE_2_ on cytotoxicity mediated by COX-2 inhibition, 1×10^4^ SK-N-BE(2) cells/well were seeded in 96-well plates. After 24 h of incubation the medium was changed and dmPGE_2_ alone or in combination with celecoxib diluted in serum-free OptiMEM medium (Gibco) supplemented with L-glutamine, penicillin G, and streptomycin, was added. After 48 h, cell viability was measured using MTT-assay. To evaluate the effect of PGE_2_ receptor antagonists on neuroblastoma cell viability, cells were incubated in 96-well culture plates with an increasing concentration of drug for 48 h. All reagents were dissolved in DMSO and further diluted in OptiMEM using the same supplements as above (the final DMSO concentration was always <0.5%).

### Statistical analysis

Two-sided unpaired t-test was performed to evaluate the accumulation of cAMP and the effect of dmPGE_2_ addition to celecoxib-treated cells. A two-way ANOVA was used to evaluate the effect of dmPGE_2_ on neuroblastoma cell viability.

## Results

### All four PGE_2_ receptor subtypes are expressed in neuroblastoma primary tumors and cell lines

**Figure 1:**
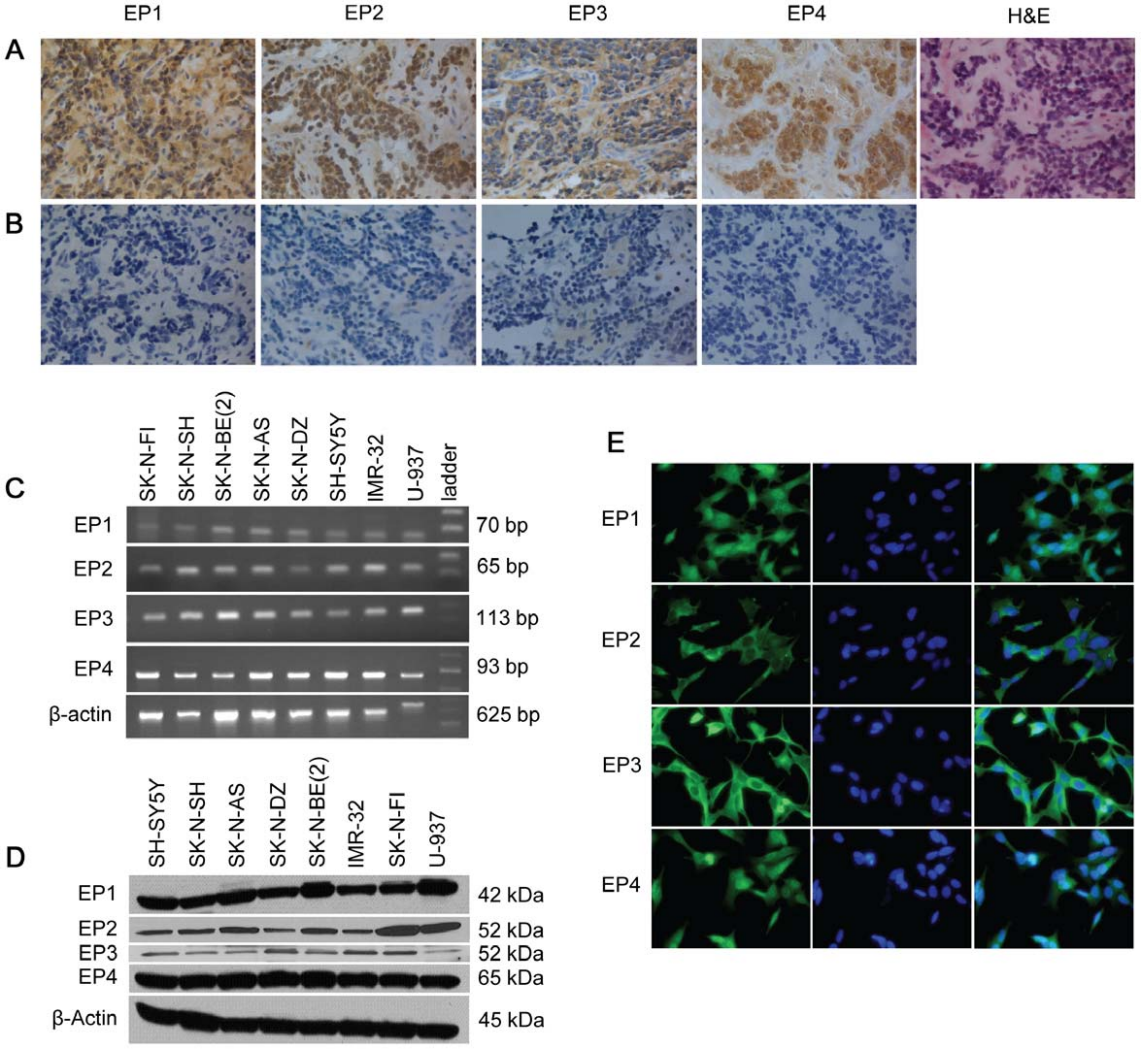
Neuroblastoma expresses all four PGE_2_ receptors. (A) Immunohistochemistry showing specific expression of EP1-4 in tumor cells of a primary human neuroblastoma. (B) Antibody specific blocking peptides were used to control for non-specific binding. Magnification ×400. (C) RT-PCR detecting EP1-4 mRNA in human neuroblastoma cell lines. The myelocytic cell line, U-937, was used as positive control. β-actin was used to ensure equal cDNA load. (D) Western blot detecting bands at 42, 52, 52 and 65 kDa corresponding to EP1, EP2, EP3 and EP4, respectively, in protein extracts from human neuroblastoma cell lines. U-937 cells were used as a positive control. β-actin was used to ensure equal protein loading. The western blots are representative of two independent experiments. (E) Immunofluorescence staining identifying EP1 and EP4 receptor expression in the cellular membrane, in the cytoplasm, and in the nuclear membrane of neuroblastoma cells. EP2 and EP3 were detected in the cellular membrane and in the cytoplasm. Cells were double stained with DAPI to visualize cell nucleus. Magnification ×400.

We stained 28 neuroblastomas from all different clinical stages and biological subsets with antibodies detecting EP1, EP2, EP3 and EP4. All tumor samples investigated showed an expression of the four receptor subtypes in tumor cells in addition to a certain degree in endothelial cells in the adjacent stromal compartment (Figure 1A). The staining was evident both in the cytoplasm and the nucleus. There was no apparent staining with any of the antibodies when pre-incubated together with respective blocking peptide, hence confirming the specificity of the antibodies using this protocol (Figure 1B). In addition, seven neuroblastoma cell lines, with different genetic aberrations and biological features were investigated and all showed expression of mRNA and protein for EP1-4 (Figures 1C and D). Further analyses of EP1-4 expression pattern by immunofluorescence revealed an expression of the receptors in cellular membranes as well as in cytoplasmic vesicles (Figure 1E). The EP1 and EP4 receptors were also shown to be present in the nuclear compartment (Figure 1E).

### Neuroblastoma cells produce PGE_2_ and dmPGE_2_ induces proliferation and survival of neuroblastoma cells

**Figure 2:**
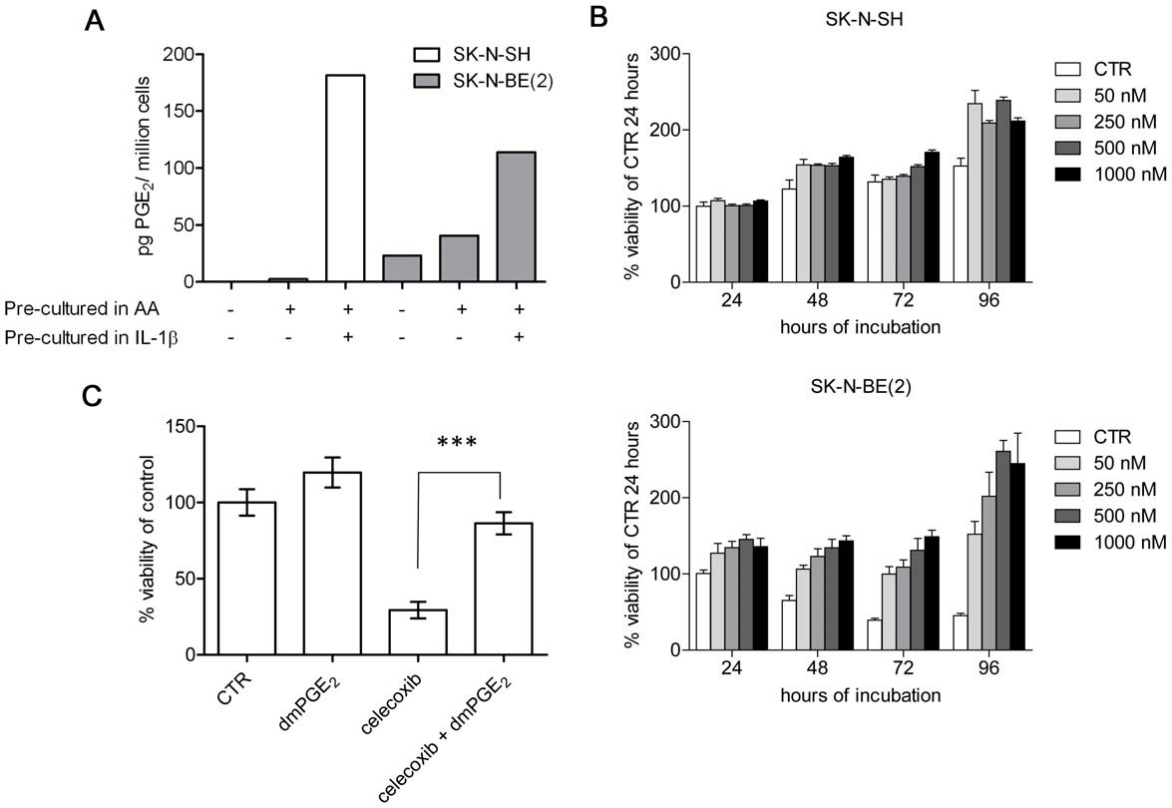
Neuroblastoma cells produce PGE_2_ and dmPGE_2_ increases cell viability. (A) Neuroblastoma cells produce PGE_2_. SK-N-BE(2) and SK-N-SH cells were cultured with or without 40 µM of arachidonic acid (AA) for 48 h and 10 ng/mL IL-1β for 12 h. Cell homogenates were incubated with 80 µM of arachidonic acid and the concentration of produced PGE_2_ was measured using LC-MS/MS. (B) PGE_2_ increases neuroblastoma cell viability. SK-N-BE(2) and SK-N-SH cells were incubated in a serum-free medium for 24 h before adding different concentrations of dmPGE_2_. Cell viability was measured using MTT-assay after 24, 48, 72 or 96 h. Values are representative of two independent experiments and data are expressed as mean (±SD) in percentage of control at 24 h. A statistical analysis was performed using 2-way ANOVA p<0.0001 for both concentration and incubation time. (C) PGE_2_ rescues neuroblastoma cells from celecoxib induced apoptosis. SK-N-BE(2) cells were incubated in 35 µM celecoxib alone or in combination with 5 µM dmPGE_2_. After 48 h cell viability was assessed using MTT-assay. Mean (±SD) of six replicate wells is shown; values are representative of three independent experiments. Statistical analysis was performed using 2-sided t test P<0.0001.

Since COX-2 and the PGE_2_ receptors are expressed in neuroblastoma, we investigated the production of PGE_2_ and its effects on cell growth. The *MYCN*-amplified, drug-resistant cell line SK-N-BE(2) produced PGE_2_ under normal culture conditions, whereas SK-N-SH cells did not produce detectable levels (Figure 2A). By culturing the cells with arachidonic acid, which is the substrate for prostaglandin biosynthesis, both cell lines were shown to produce PGE_2_ and stimulation with IL-1β further increased the production (Figure 2A). The addition of dmPGE_2_ to serum-starved SK-N-BE(2) and SK-N-SH cells resulted in an increased cell viability and proliferation in a dose-, time- and cell line-dependent manner (p<0.0001, Figure 2B). The effect was more pronounced for the SK-N-BE(2) cells because of the reduced cell growth of serum starved controls. We have previously shown that the inhibition of COX-2 with celecoxib, at concentrations above 28 µM, significantly reduces neuroblastoma cell viability [3]. To investigate whether exogenously added dmPGE_2_ could rescue neuroblastoma cells from celecoxib-induced toxicity we treated SK-N-BE(2) cells with 35 µM celecoxib alone or in combination with 1 µM of dmPGE_2_. As shown in Figure 2C, dmPGE_2_ completely abrogated celecoxib-mediated cytotoxicity (p<0.0001).

### Addition of dmPGE_2_ increases the intracellular concentrations of calcium and cAMP with a subsequent phosphorylation of Akt in neuroblastoma cells

**Figure 3:**
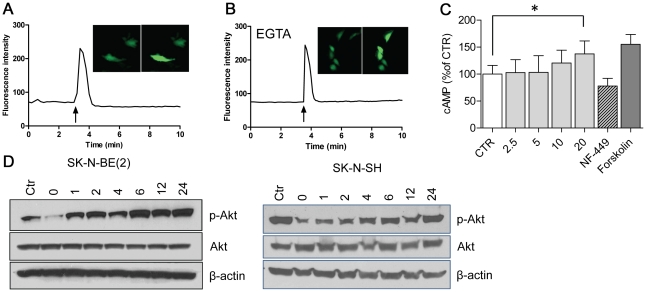
dmPGE_2_ increases intracellular Ca^2+^ and cAMP concentrations followed by phosphorylation of Akt. (A) Intracellular calcium mobilization in response to dmPGE_2_. SK-N-SH cells were loaded with the calcium fluorescent dye Fluo-4/AM before the addition of 1 µM dmPGE_2_ or (B) pre-treatment with 2 mM EGTA before exposure to 1 µM dmPGE_2_. The fluorescence intensity was followed using a confocal laser scanning microscope and representative single-cell recordings are shown. The arrows indicate when dmPGE_2_ is added. (C) Intracellular accumulation of cAMP in response to dmPGE_2_. SK-N-SH cells were incubated overnight in a medium without serum before the addition of 1 µM of dmPGE_2_. Pretreatment with 10 µM NF 449, which is a Gαs inhibitor, before the incubation in dmPGE_2_ for 10 min inhibited the production of cAMP. Forskolin, 10 µM for 10 min, was used as a positive control. The graph shows mean (±SD) in % of untreated control of three independent experiments. A statistical analysis was performed using 2-sided t-test, P<0.05. (D) PGE_2_ induces phosphorlyation of Akt. SK-N-BE(2) and SK-N-SH cells were grown in the presence of serum (Ctr) before 24 h of culturing in the absence of serum (0 h) prior to the addition of 1 µM of dmPGE_2_. Cells were further incubated in dmPGE_2_ for 1, 2, 4, 6, 12 or 24 h and protein extracts were subjected to western blotting to detect phosphorylated Akt(ser473). An antibody detecting unphosphorylated Akt was used to exclude differences in total protein expression. β-actin was used to control for equal protein loading. The western blots are representative of three independent experiments.

EP1-4 are coupled to G-proteins that activate various second messengers and signaling cascades. In the responding SK-N-SH cells, the addition of dmPGE_2_ resulted in a rapid increase in the cytoplasmic level of Ca^2+^. Pre-incubation with the calcium chelator EGTA did not alter the response, thus demonstrating that Ca^2+^ is released from intracellular stores (Figure 3A and B). The addition of dmPGE_2_ to SK-N-SH cells also resulted in a significantly increased concentration of cAMP after 20 min of incubation (p<0.05, Figure 3C). The concentration was in the same range as after 10 min of incubation with forskolin, known to specifically activate adenylate cyclase. In addition, the Gαs inhibitor NF 449 inhibited dmPGE_2_-mediated production of cAMP. Moreover, the addition of dmPGE_2_ to serum-starved SK-N-SH and SK-N-BE(2) cells resulted in a sustained phosphorylation of Akt(ser 473), (Figure 3D).

### Inhibition of PGE_2_ receptor signaling reduces neuroblastoma cell viability

To evaluate the importance of each of the PGE_2_ receptor subtypes for neuroblastoma cell viability we incubated cells with a panel of receptor antagonists (Table 1). Inhibition of the EP1 receptor resulted in a 50% reduced cell viability (EC_50_) at 8–20 µM of the non-prostanoid compound ONO-8713, and at 35–50 µM of SC-51322, which has been widely used for EP1 receptor characterization [15]. To the best of the authors knowledge, specific receptor antagonists for EP2 are not commercially available, however, the widely used AH6809 has an affinity not only for the EP2 receptor but also for EP1 and the PGD_2_ receptor 1, and the selectivity is poor at concentrations in the micromolar range [15]. The EC_50_ value on neuroblastoma cell viability for AH6809 ranged from 30 µM to >100 µM. Incubation with the highly selective EP3 receptor antagonist, ONO-AE3-240, resulted in an EC_50_-value of 7.5–22.5 µM. We also treated cells with two different EP4 receptor antagonists and the EC_50_ range was 10–20 µM and 30 to >100 µM, for L-161,982 and AH23848, respectively.

**Table 1:** EC_50_ of EP1-4 receptor antagonists on neuroblastoma cell viability *in vitro*.

EC_50_ (µM)							
Drug	Drug target	SK-N-SH^b^	SH-SY5Y	SK-N-AS^b^	SK-N-FI^b^	IMR-32^a^	SK-N-BE(2)^a^^,^ b
ONO-8713	EP1	12–14	12–14	12–14	18–20	10–12	8–10
SC-51322	EP1	35–40	35–40	40–45	45–50	40–45	35–40
AH6809	EP1, EP2, DP1	45–60	30–45	>100	60–75	45–60	75–90
ONO-AE3-240	EP3	10–12.5	7.5–10	12.5–15	20–22.5	12.5–15	7.5–10
AH23848	EP4, TP	40–50	30–40	>100	60–70	30–50	70–80
L-161,982	EP4	10–15	5–10	10–15	15–20	10–15	10–15

Abbreviations: EC_50_; effective concentration decreasing neuroblastoma cell viability with 50%,

a MYCN amplification;

b Multidrug-resistant phenotype.

## Discussion

Upregulation of COX-2 and an increased PGE_2_ level are frequently detected in premalignant and malignant tissues of epithelial origin in adults. Several experimental, epidemiological and clinical studies suggest that COX inhibitors have a potential as chemopreventive therapy by reducing chronic inflammation that predispose cancer development [1]. Further studies have revealed that PGE_2_ possess various of important effects attributed to tumor growth such as increased proliferation, angiogenesis, metastasis and immune suppression [1]. Whether PGE_2_ exerts the same effects in childhood tumors is not yet clear. Recent therapeutic phase III studies in adults, using COX- 2 inhibitors in an adjuvant setting of colorectal cancer and non-small cell lung cancer, showed no significant survival benefit [16], [17]. Therefore, additional studies investigating the link between PGE_2_ and cancer and the potential of a more specific targeting of prostaglandin signaling are needed. We have previously reported high COX-2 expression in neuroblastoma, a childhood tumor of the sympathetic nervous system, and medulloblastoma, in the central nervous system, and showed a pronounced effect with COX inhibitors in treatment *in vivo* [3], [5], [18]. However, no studies have addressed the specificity of COX-2 inhibition in neuroblastoma or the function of its major end product PGE_2_.

By immunohistochemistry, we detected expression of all four PGE_2_ receptor subtypes, EP1, EP2, EP3 and EP4, in the tumor cells of all primary neuroblastomas investigated, independent of any biological characteristics or clinical stage (Figures 1A and B).The receptors were also expressed in the vasculature of the adjacent stromal tissue. Furthermore, mRNA and protein corresponding to EP1-4 were found to be expressed in all seven neuroblastoma cell lines, exhibiting different genetic aberrations and biological features ([19], Figures 1C and D). The EP2 receptor has previously been shown to be epigenetically silenced in preferentially *MYCN*-amplified neuroblastoma tumors and cell lines, and therefore suggested to act as a tumor suppressor in aggressive neuroblastomas [20]. We could not find this suggested correlation in either our clinical patient material including several tumor samples with *MYCN* amplification, or cell lines derived from high-risk patients with or without *MYCN* amplification. In addition, when using the publicly available R2 microarray analysis and visualization platform with expression array data from 88 neuroblastoma tumors (available at http://r2.amc.nl [21],) EP2 mRNA expression was significantly correlated with poor clinical outcome with highest expression found in *MYCN*-amplified tumors (data not shown). An increased EP receptor expression has been reported in medulloblastoma, and in various adult epithelial cancers. Many tumors show differential expression of the EP receptors and the expression pattern seems to be dependent on the tumor type [18], [22]–[28].

In addition to the conventional restriction of GPCR expression to the cell membrane, the PGE_2_ receptors have been reported to be localized in the nuclear membrane and we therefore investigated the cellular localization of the receptors in neuroblastoma cells by immunofluorescence [10], [29]. We detected EP1-4 in the cellular membrane and in vesicles in the cytoplasm (Figure 1E). Besides, EP1 and EP4 were also shown to be present in the nuclear compartment (Figure 1E). The presence of nuclear EP receptors suggests that in addition to a conventionally autocrine or paracrine manner, PGE_2_ may be capable of acting intracellularly, thereby potentially influencing nuclear events [30]. Interestingly, EP1 is expressed both in the cytoplasm and nucleus of breast cancer cells and a nuclear expression of EP1 is associated with a better prognosis for these patients [31].

We also demonstrate that neuroblastoma cells are able to produce PGE_2_, and that incubation with arachidonic acid or IL-1β increases the production (Figure 2A). These results show a functional COX pathway that can be triggered by inflammatory stimuli, suggesting a prominent role for COX-2 in the production of PGE_2_ in neuroblastoma cell lines. Importantly, PGE_2_ is a potent proinflammatory molecule involved in the crosstalk between tumor cells and infiltrating immune cells. In the tumor microenvironment, production of PGE_2_ by neuroblastoma cells may contribute to inflammation and immune suppression [1]. To further explore the effect of PGE_2_ on neuroblastoma cell viability, dmPGE_2_ was added to serum-starved neuroblastoma cell cultures that stimulated neuroblastoma cell proliferation and survival in a dose-, time- and cell line-dependent manner (Figure 2B). These results extend our earlier study demonstrating a dose-dependent effect on the proliferation of medulloblastoma cells [18]. The toxic effect of celecoxib was also attenuated by dmPGE_2_ suggesting an importance of PGE_2_ in COX-2 mediated signaling in neuroblastoma (Figure 2C).

Given the fact that neuroblastoma cells produce PGE_2_ as well as the complexity that follows the expression of all four receptor subtypes, we decided to investigate the effect of exogenously added dmPGE_2_ on intracellular signaling pathways. Signal transduction downstream of the different EP receptor subtypes has been studied by agonist-induced alterations of second messengers and these studies indicate that the receptors do not exclusively couple to one G-protein and one downstream signaling pathway [11]. In addition, not only the Gα subunit is important in cell signaling, as the βγ subunits also interact with and stimulate downstream effectors [32]. In neuroblastoma, dmPGE_2_ increased the cytoplasmic Ca^2+^ level by its release from intracellular Ca^2+^ stores. We also detected a significant increase in cAMP which was inhibited by pre-treatment with a Gαs inhibitor. These results indicate that neuroblastoma cells respond to PGE_2_ by activation of more than one EP receptor, followed by a subsequent stimulation of an intracellular Ca^2+^ release and production of cAMP. Calcium release is predominantly a result of the activation of EP1, but due to the different splice variants of the EP3 receptor, this receptor is capable of coupling with several different G-proteins including Gαq which stimulates Ca^2+^ mobilization [33]. The stimulation of adenylate cyclase and cAMP is primarily a result of the activation of EP2 and/or EP4. The EP3 receptor has also been shown to be able to couple to Gαs, whereas the major splice variant couples to Gα_i_, thus inhibiting cAMP production [11]. Calcium is a ubiquitous second messenger and its cellular concentration and distribution are strictly regulated. In cancer, Ca^2+^ has been implicated in several important features of tumorigenesis including motility, angiogenesis, transcription, differentiation, cell cycle regulation and apoptosis [34]. Moreover, Ca^2+^ activates cPLA_2_ that subsequently releases arachidonic acid [35]. The effect of cAMP is mediated by cAMP-dependent protein kinase A (PKA) and the exchange proteins activated by cAMP (EPACs). The role of cAMP-PKA and -EPACs in cancer appears to be dependent on the cell type. The activation of these proteins has been shown to either stimulate cell proliferation, or reduce cell growth and promote differentiation [36], [37]. The role of cAMP in neuroblastoma is not fully elucidated, though it has been shown that neuroblastoma cells contain a low level of cAMP and that the treatment with a cAMP analog induces differentiation *in vitro* [38]. Furthermore, the production of cAMP in response to PGE_2_ is increased in retinoic acid-differentiated SK-N-BE(2)C cells [39].

The activation of G-proteins initiates further intracellular signaling, which is mediated either by Gα and a change in second messengers or by the βγ subunits. In epithelial cancers, PGE_2_ has been shown to activate important signaling pathways involved in proliferation and survival such as PI3K/Akt, MAP-kinases, Wnt and transactivation of EGFR [1]. In neuroblastoma, the majority of primary tumors exhibit a phosphorylation of Akt and a high degree of phosphorylation correlates with a poor prognosis [40], [41]. We and others have shown that inhibition of Akt signaling induces apoptosis and reduces neuroblastoma tumor growth [40]–[44]. These studies reveal that Akt is a promising novel target for neuroblastoma therapy [45]. The addition of dmPGE_2_ to serum-starved neuroblastoma cell cultures, resulted in an increased phosphorylation of Akt (Ser473) that was sustained after 24 h of incubation (Figure 3D). The activation of Akt can trigger many downstream signaling cascades, and in colon cancer PGE_2_ has been shown to activate Akt and the downstream pathway of GSK-3β and β-catenin resulting in an increased proliferation, whereas an increased survival was due to Akt activation of PPARδ [46], [47]. Activation of the Akt signaling pathway could explain the mechanism underlying the positive effect of PGE_2_ on neuroblastoma cell viability.

We also investigated the effect of PGE_2_ receptor antagonists on neuroblastoma cell viability (Table 1). The most effective drugs in reducing cell viability were ONO-8713, ONO-AE3-240, and L-161,982 targeting the EP1, EP3 and EP4 receptors, respectively. These results suggest that more than one receptor is important for neuroblastoma cell viability. However, the results are also dependent on differences in specificity and affinity of the respective antagonist for the receptor [15]. The complex expression pattern of the PGE_2_ receptors in neuroblastoma, as well as in other cancers, makes it difficult to dissect the importance of a specific receptor. Several studies indicate that the receptors may play different roles in tumorigenesis [48]–[50]. A study using the *Apc^1309^* mouse model shows that treatment with the EP1 antagonist, ONO-8711, reduces the number of intestinal polyps whereas treatment with an EP4 antagonist results in smaller polyp size. Furthermore, treatment with the two antagonists in combination shows an additive effect on the number and size of intestinal polyps [51].

Taken together, our results demonstrate that PGE_2_ is produced in neuroblastoma and that the PGE_2_ receptors are abundantly expressed, hence constituting an autocrine and/or paracrine survival loop of significance for tumor growth and spread of this childhood tumor. Thus, strategies based on selective targeting of PGE_2_ signaling could be an alternative therapeutic approach against this cancer that could avoid the otherwise potential side effects of COX-2 inhibitors.
